# Supplementary figures and images for: Generation of a Microsporidia Species Attribute Database and Analysis of the Extensive Ecological and Phenotypic Diversity of Microsporidia
Source: mBio. 2021 Jun 29;12(3):e01490-21. doi: 10.1128/mBio.01490-21 (PMC8262960; doi:10.1128/mBio.01490-21)

# Arthropoda Order

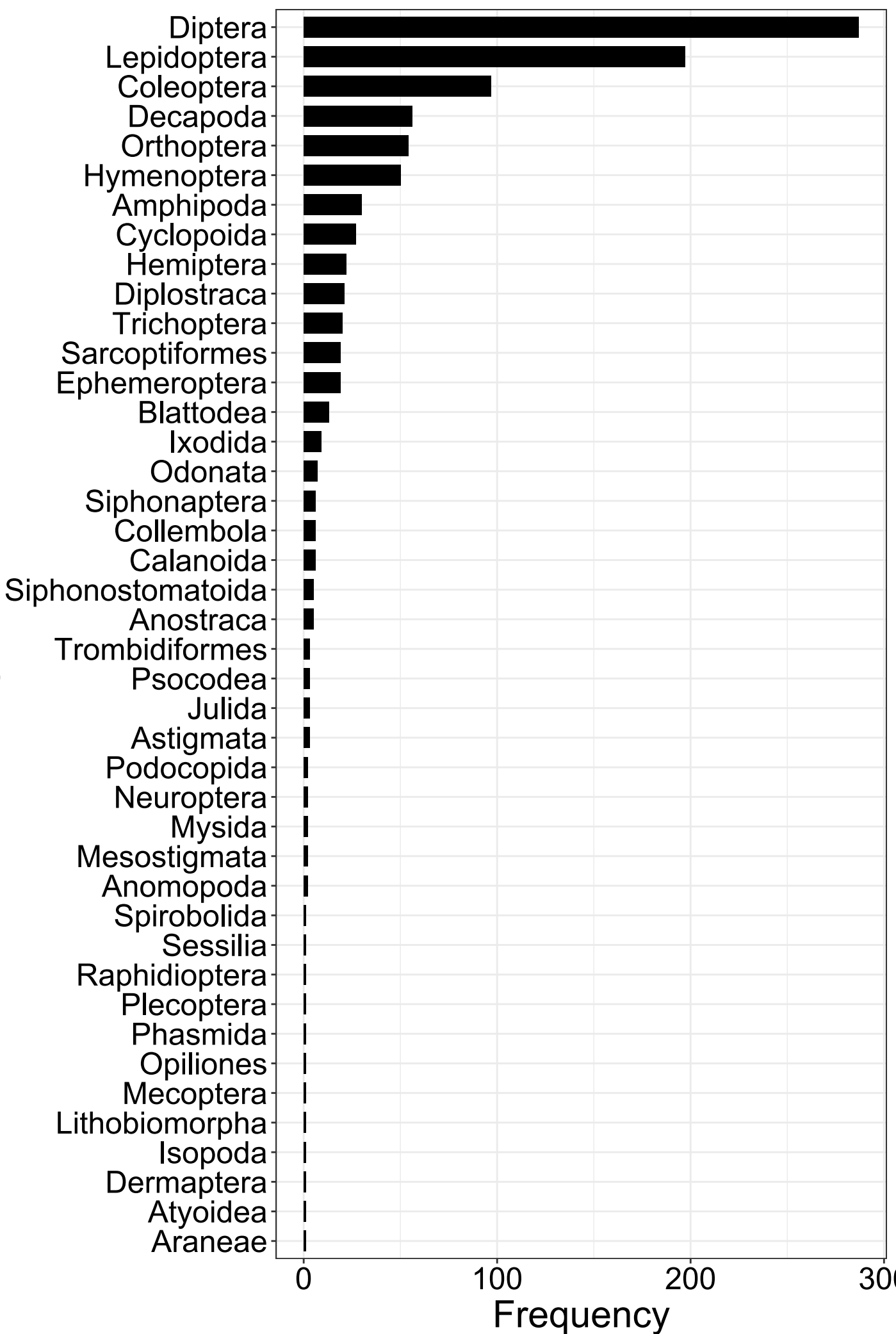

Supplement: FIG S1 [file mbio.01490-21-sf001.pdf]

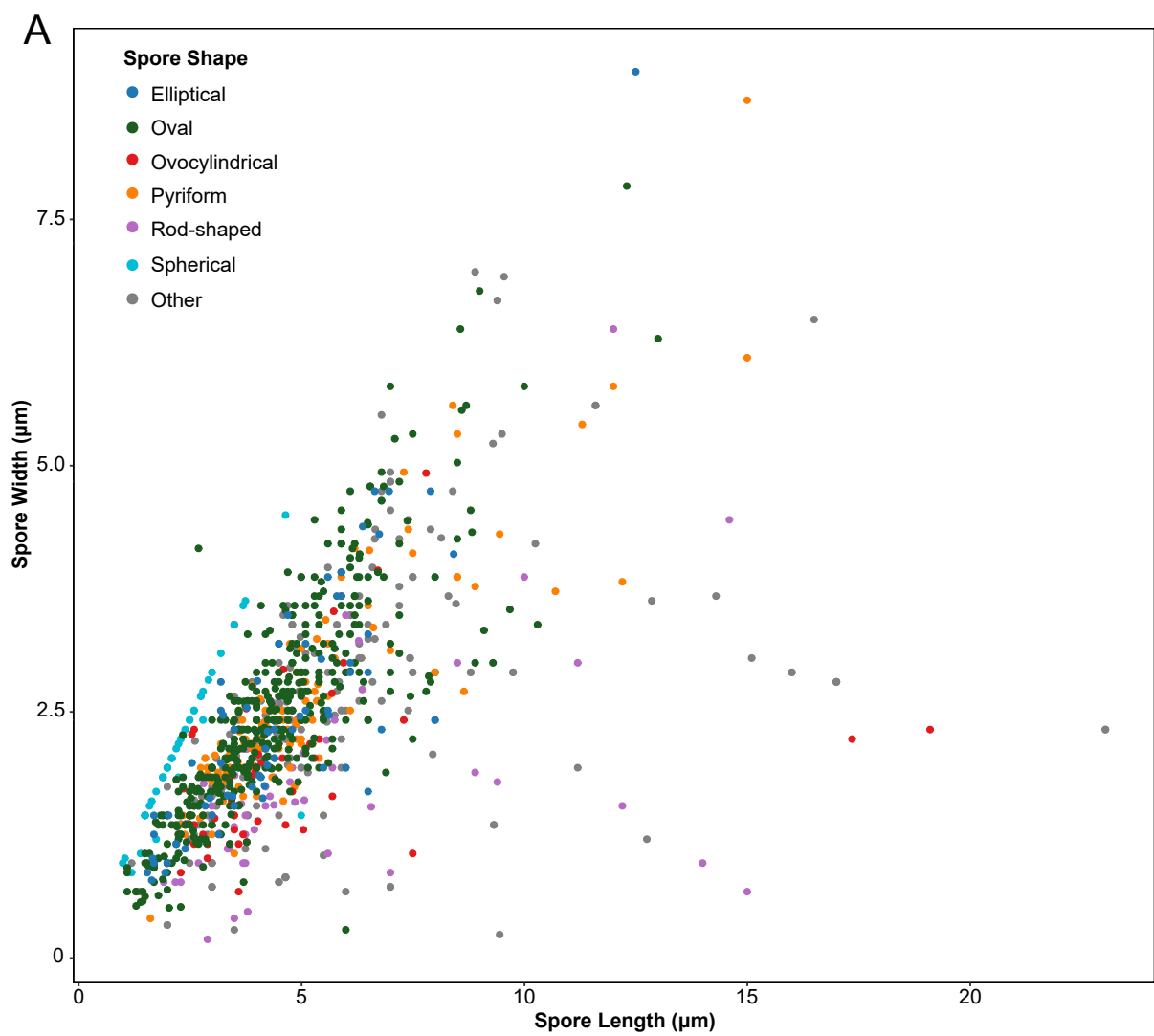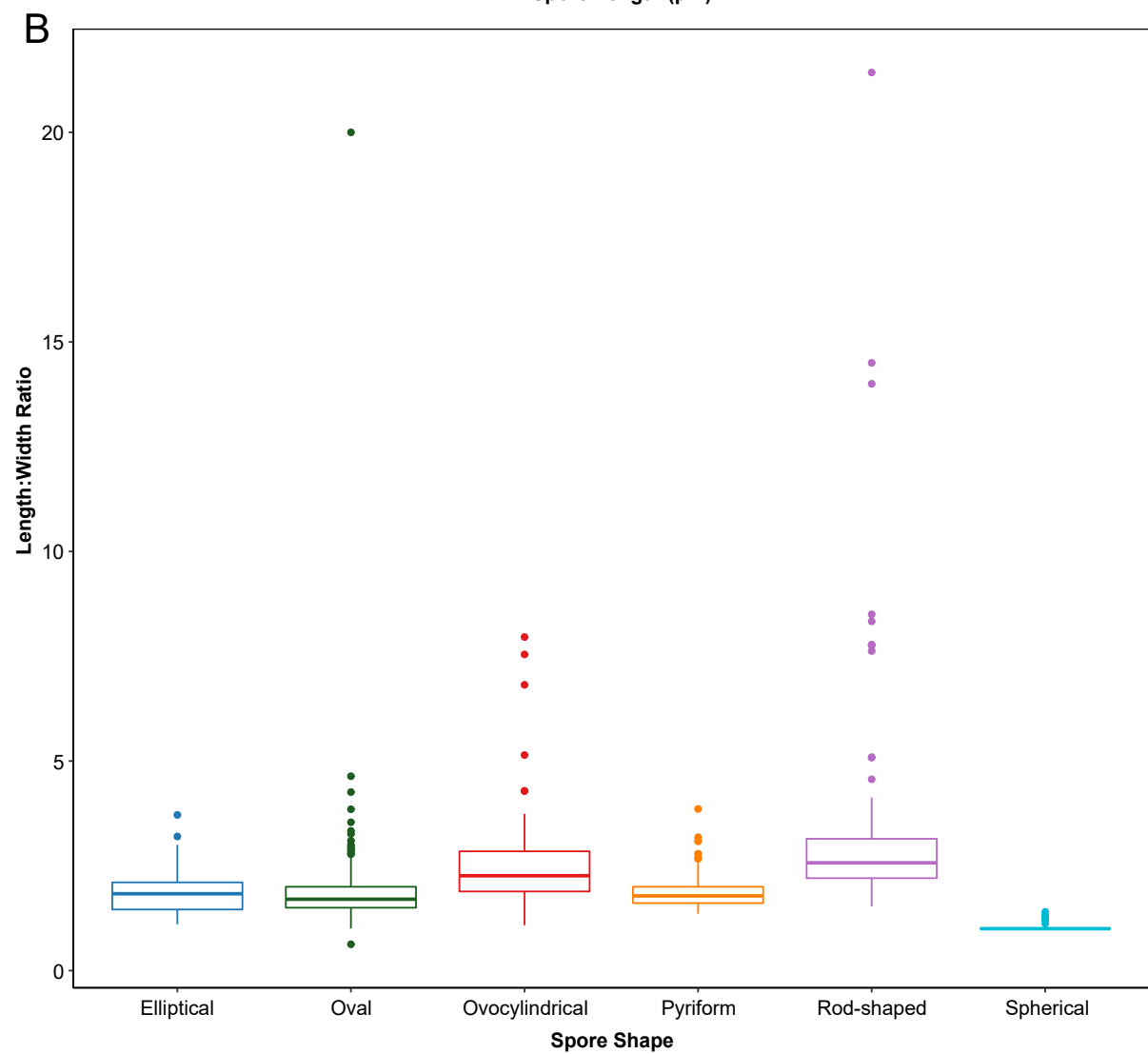

Supplement: FIG S2 [file mbio.01490-21-sf002.pdf]

A

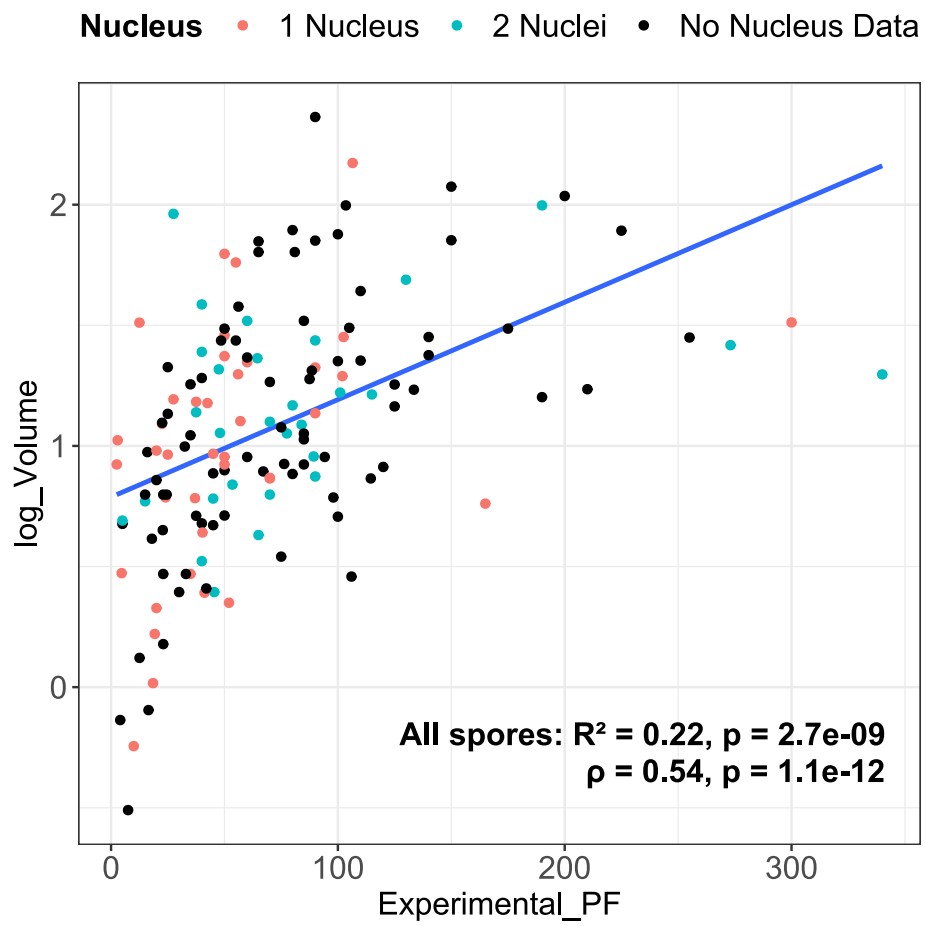

B

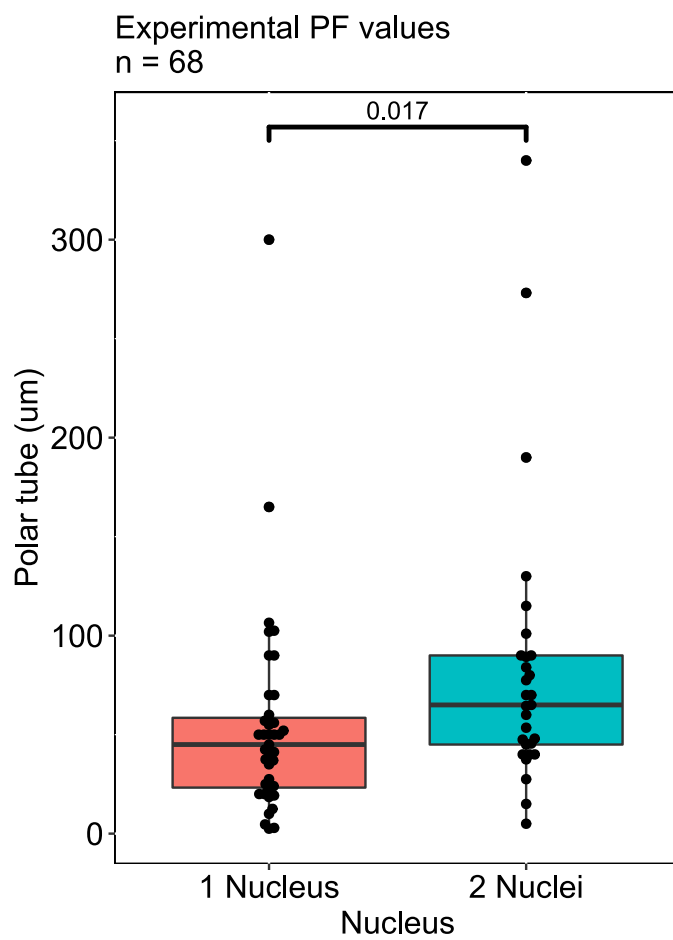

Supplement: FIG S3 [file mbio.01490-21-sf003.pdf]

**A**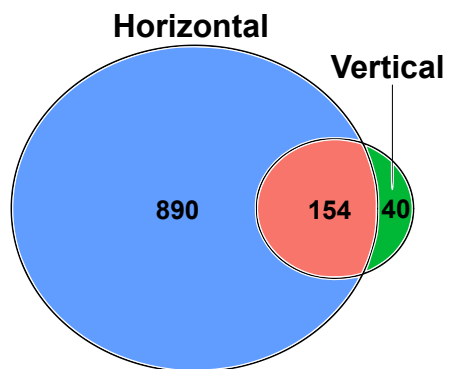**B**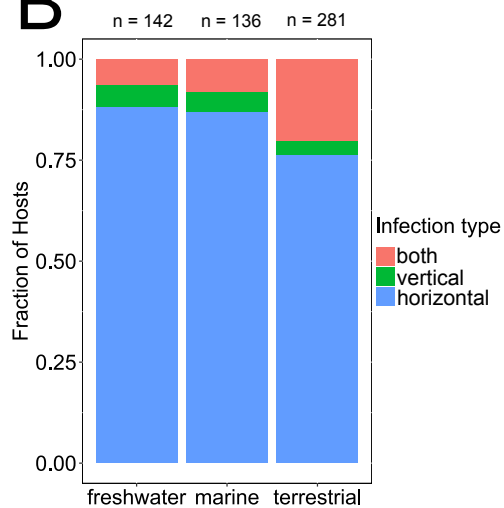**C**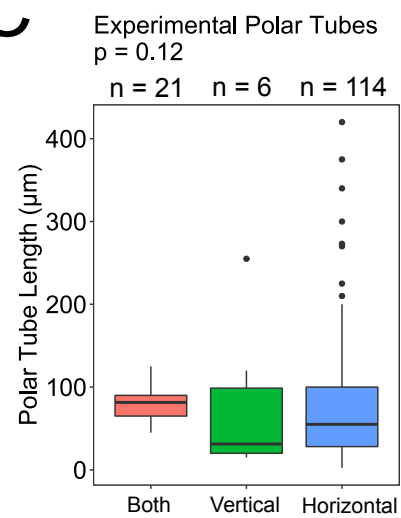**D**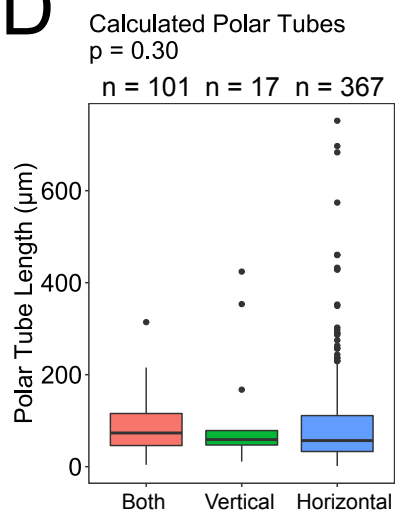**E**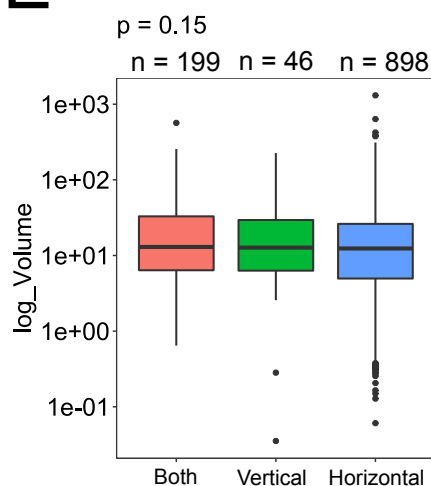

Supplement: FIG S4 [file mbio.01490-21-sf004.pdf]

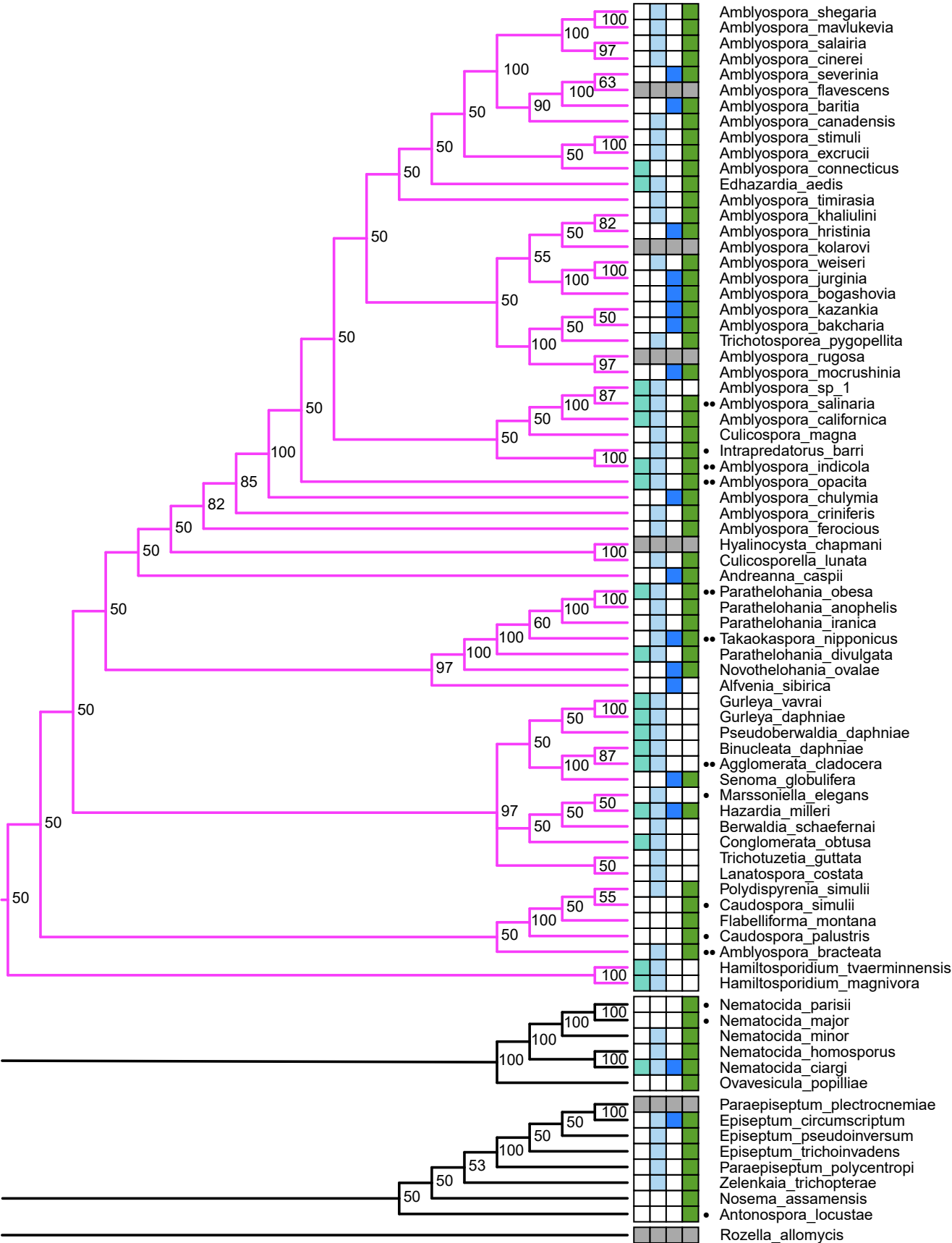

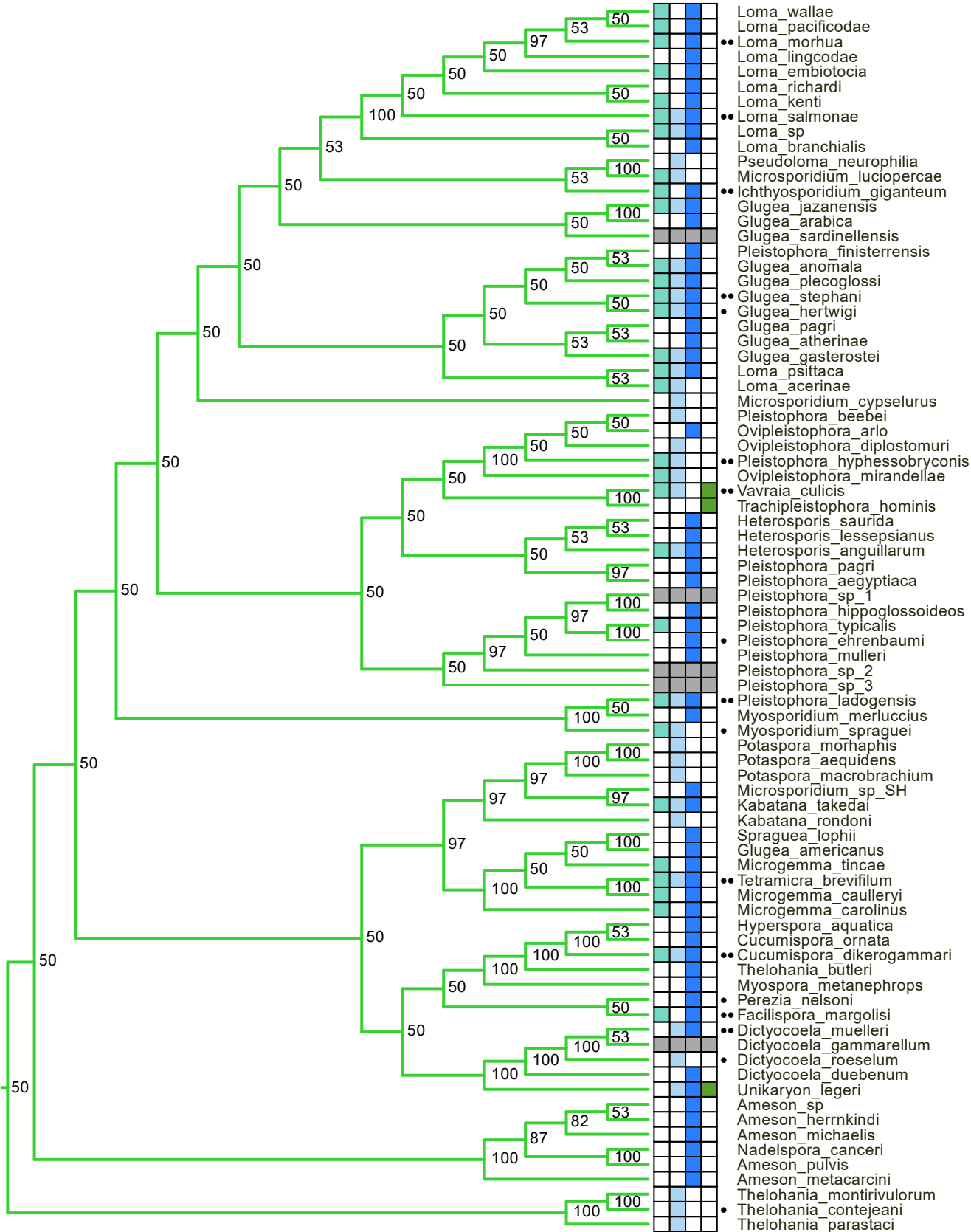

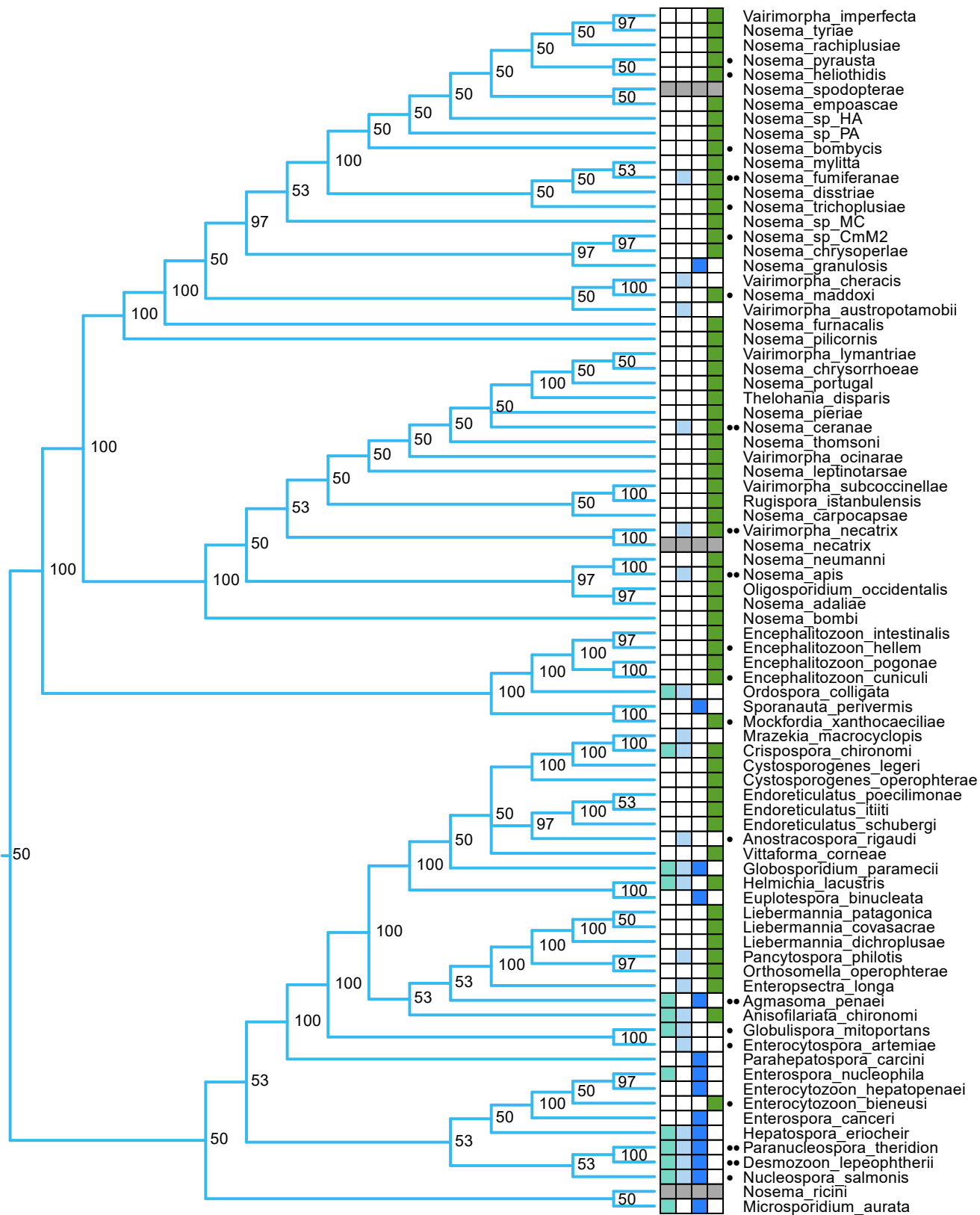

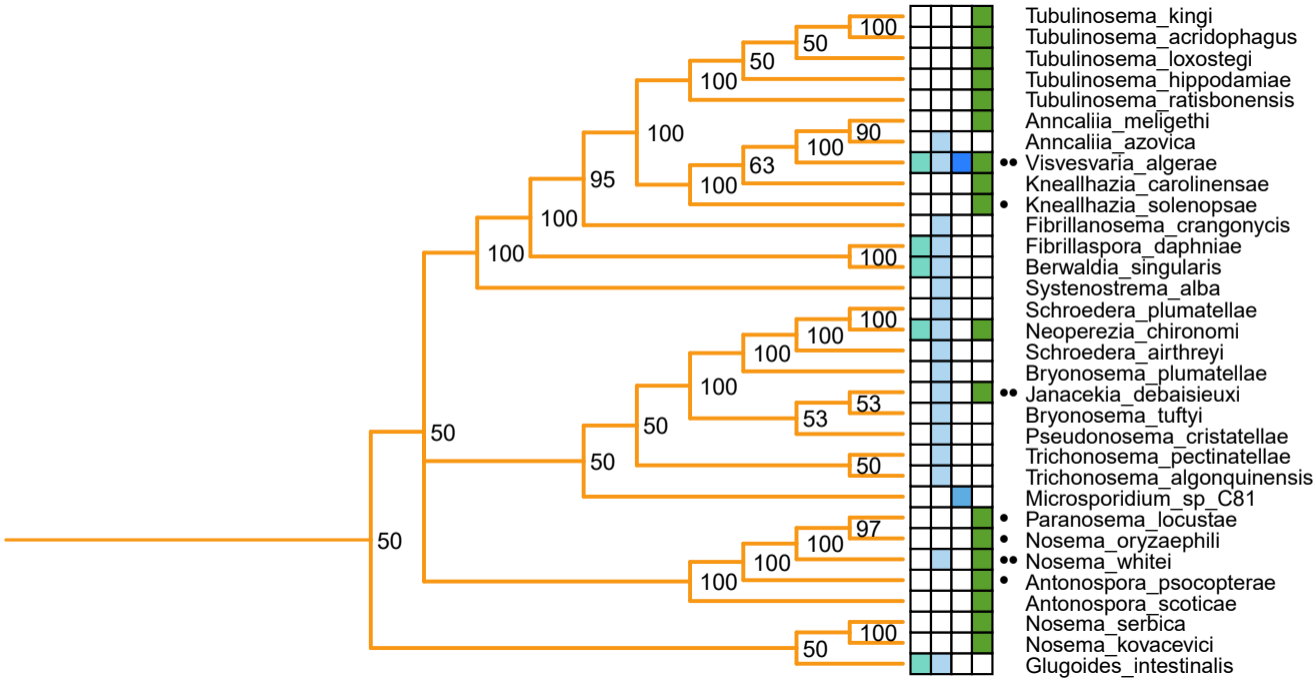

Supplement: FIG S5 [file mbio.01490-21-sf005.pdf]
